# Therapeutics

**Published:** 1859-05

**Authors:** 


### THERAPEUTICS.

1. On the Use of Colchicum in Gout. By *Dr. Günsburg, of Breslau.*—As yet there being little known of the effects of colchicum, the following notice, communicated by Dr. Günsburg, may prove interesting. The author has used colchicum for five years in numerous cases. To older patients who had suffered for a long time from gout, he ordered, during the painful paroxysms of swelling of the joints, one-sixtieth of a grain of the alkaloid three times daily. The remedy always acted as an intense stimulus to the intestinal secretion, even in such patients who were subject to constipation. After using the remedy from three to four weeks, patients who otherwise every few months suffered an attack, remained free from it for more than a year. Many a gouty person was thus saved from passing a painful and troublesome winter in the bed or easy chair. Dr. Günsburg has not yet tried the remedy in cases in which the articular swelling is accompanied by inflammation of the skin. Against all expectations colchicum proved of but little use in acute articular rheumatism, and after repeated trials Dr. Günsburg desisted from using it in cases of this kind.—(*Zeitschrift für Klin. Medizin.*, 1858.)

2. GLYCERIN IN CHRONIC LARYNGITIS. By *Dr. Posner.*—In cases of chronic laryngitis in which patients complain of constant heat and dryness, and an uneasy tickling sensation in the throat, with continued inclination to cough, Dr. Posner of Berlin recommends the application of ten to fifteen drops of glycerin every hour, of which he has seen the most surprising results. He attributes the efficacy of the remedy to the circumstance that it lessens the irritation of the diseased mucous membrane by covering it with an emollient coat.—(*Medizinische Central Zeitung*, September, 1858.)

3. GLYCERIN IN HYPERÆSTHESIA VULVÆ, ERYTHEMA NODOSUM, AND VAGINITIS. By *M. Paupert.*—1. M. Paupert used glycerin with excellent success in a case of *hyperæsthesia vulvæ*, accompanied by an eruption of ecthyma. The patient, a lady forty years of age, had suffered from this disease for several years, and had been under medical treatment all the time without obtaining relief. M. Paupert introduced compresses soaked in glycerin, into the vagina, twice a day, and ordered cold ablutions every morning and evening. The pruritus, as well as the ecthyma pustules, disappeared very soon; the cure was accomplished about three years ago, and the patient has had no relapse.

2. A man, twenty-eight years of age, suffered of *erythema nodosum* and general hyperæsthesia of the skin, in consequence of imprudent hydropathic treatment. The skin was dry, and its functional activity very feeble. The disease had become so vehement that even the mental faculties of the patient were disturbed to a high degree. M. Paupert ordered the whole body to be rubbed with glycerin twice a day, and after this treatment had been continued for several weeks, the itching and the eruption disappeared, the skin resumed its normal functions, and the patient was dismissed cured.

3. In vaginitis, with superficial ulceration of the collum uteri, a combination of glycerin (ʒj) with tannic acid, (grs iv.) which the author calls *glycerole de tannin*, has proved very useful; it removed within a short time the inflammatory symptoms, and promotes cicatrization of the ulcers.—(*Journ. de Méd. de Bruxelles*, Sept. 1858.)

